# Analysis of Potential Risks Associated with Urban Stormwater Quality for Managed Aquifer Recharge

**DOI:** 10.3390/ijerph16173121

**Published:** 2019-08-27

**Authors:** Yalin Song, Xinqiang Du, Xueyan Ye

**Affiliations:** 1 Key Laboratory of Groundwater Resources and Environment, Ministry of Education, Jilin University, Changchun 130021, China; 2 College of New Energy and Environment, Jilin University, Changchun 130021, China

**Keywords:** managed aquifer recharge, urban stormwater, contamination risk, clogging

## Abstract

Managed aquifer recharge (MAR) can be used to increase storage and availability of groundwater resources, but water resources available for recharge are constrained due to a surface water shortage. Alternative resources, like stormwater, are receiving increasing attention as sustainable resources for reuse in MAR. However, pollutants in stormwater can impact groundwater quality, and cause clogging of the infiltration system. Based on the stormwater data in the literature, the physicochemical stormwater properties of data were analyzed. The results showed that concentrations of pollutants from different underlying surfaces varied widely. The main pollutants of stormwater were as follows: Total suspended particles (TSSs), organic matter represented by the chemical oxygen demand (COD), nutrients (total nitrogen, TN; total phosphorus, TP; and NH_3_-N), and metals (Zn, Pb, Cu, Cd, Fe, and Mn). Based on the simulation of TOUGHREACT, the contamination risk of pollutants for each type of stormwater was assessed. The risk of contamination was divided into four categories due to the different migration times of ions through the sand column. The iron ion has the highest risk of contamination, followed by Zn and Mn, and the contamination risk of nutrients and other metals (Pb, Cu, and Cd) are relatively low. Besides, the physical, biological, and chemical clogging risk were evaluated. The physical clogging potential of all types of stormwater is very high because of the high concentration of TSS. According to the concentration of TN that can spur the growth of bacteria and algae, the relative risk of biological clogging for stormwater is greenbelt stormwater < road stormwater < roof stormwater. However, only road stormwater has high chemical clogging due to the existence of iron, which can generate precipitation that blocks the pore volume.

## 1. Introduction

With the booming of the population and economy, global urbanization has accelerated, especially in developing countries. The world’s population in urban areas has increased from 1332 million in 1970 to 3495 million in 2010, with an annual growth rate of 2.4% [1]. In China, the urbanization rate increased from 10.64% in 1949 to 52.57% in 2012, with an annual growth rate of 2.6% [2]. Due to rapid urbanization processes, impervious surfaces are continually spreading, and this leads to an increase in the runoff water volume and water quality degradation. The highest percentage of land surface in urban areas is covered by rooftops and pavement. Moreover, rapid urbanization has triggered a series of hydrological effects [3]. On the other hand, as the groundwater supply decreases, the problem of groundwater over-exploitation becomes increasingly widespread [4].

Managed aquifer recharge (MAR) is the purposeful recharge of water to aquifers for subsequent recovery or environmental benefit [5]. MAR can not only be used to increase the storage and availability of groundwater resources but can also reduce the risk of seawater intrusion and land subsidence, and improve water quality [6,7,8]. However, many MAR projects are restricted by the shortage of water resources that could be tapped into for groundwater recharge. Traditional recharge water sources include the surface water of rivers, lakes, or reservoirs, and ground water from other aquifers. In recent years, the utilization of reclaimed water has been consistently increasing. MAR with reclaimed water plays a particular role due to both the large potential benefits achieved in terms of sustainable water resource management as well as the complexity of the planning and implementation of such schemes [6,9,10,11,12]. On the other hand, stormwater is receiving significant attention as a viable alternate water resource for reuse, which is currently under-utilized [13].

Currently, stormwater in Australia, the United States, India, and other countries is increasingly used as a recharge source [5,14]. According to a survey done in Australia, the public has a high degree of acceptance for artificial recharge with stormwater [15]. Compared to surface water and reclaimed water, the availability of urban stormwater mainly depends on the quantity and spatial and temporal distribution of rainfall. Moreover, centralized stormwater treatment is a significant challenge for the use of urban stormwater since it may affect the groundwater quality [16]. At the same time, MAR by stormwater is often limited by clogging that makes the recharging rate too slow. Therefore, the key to the feasibility of urban stormwater should depend on the risk of groundwater pollution, followed by the risk of clogging during the MAR process. Clogging is an inevitable problem that is recognized as perhaps the most significant challenge in MAR operations; it is attributed to physical, chemical, and biological processes.

In this study, based on a data set of pollutant concentrations of stormwater runoff from different underlying surfaces, the general characteristics of the pollutants were determined, and then the key factors that affect the concentration of pollutants were analyzed. Moreover, the pollution potential and clogging risk posed by stormwater were assessed, and suggestions on stormwater reuse are given.

## 2. Materials and Methods

### 2.1. Stormwater Quality Data

The pollutant concentration in stormwater usually varies depending on the type and material of the underlying surface of the city [17,18]. The urban stormwater data used in this study were mainly collected from the literature, especially from studies that tested a variety of substances. In order to facilitate the analysis of water quality data, on the basis of the literature collection, these indicators, which were tested more frequently, were classified and summarized. These indicators included total suspended solids (TSSs), nutrients (total nitrogen, TN; total phosphorus, TP; NH_3_-N), organic matter (represented by chemical oxygen demand, COD), and metals (Zn, Pb, Cu, Cd, Fe, and Mn). Also, the urban stormwater data was classified into three types (greenbelt, road, and roof runoff) due to their distinct characteristics.

#### 2.1.1. Road Stormwater Runoff Quality

Due to the high impermeability of urban roads, pollutants are dissolved, washed, and moved into the water body through the urban discharge pipe network during periods of rainfall, causing significant degradation to the water quality of the receiving water body [19]. With frequent traffic activity, road stormwater runoff can accumulate large amounts of suspended particulates and nutrients, as shown in Table 1. Besides, heavy metal pollution originating from traffic activities is also significant. Because of the erosion and dissolution of stormwater water, the heavy metal generated by the accumulated traffic on the road surface is loaded into the runoff. Traffic activities that cause heavy metal pollution mainly include motor vehicle exhaust emissions, fuel dripping, brake and tire wear, road degradation, road debris, and road maintenance [20,21,22]. Heavy metal concentrations in the road runoff of cities are shown in Table 2.

#### 2.1.2. Roof Stormwater Quality

There are two main contamination sources of stormwater from roof surfaces. One is particles, which can accumulate on the roof surface either from direct atmospheric deposition or from overhanging foliage or bird and rodent droppings. Alternatively, roof material itself continuously degrades and can contribute both particulate matter and dissolved metals to runoff water [55]. 

There are three roof materials: Tile, asphalt, and cement. The concentration of pollutants from various roof types is shown in Table 3. Moreover, based on past studies, heavy metal pollutants in roof stormwater make a large contribution to total urban stormwater quality [56,57]. The collected concentrations of heavy metal ions in the stormwater of urban roofs are shown in Table 4.

#### 2.1.3. Greenbelt Stormwater Quality

The greenbelt is an important organic component of the city, which can play an important role in beautifying the environment, reducing rainfall runoff, and replenishing groundwater. During a typical rainfall process, some of the pollutants will be intercepted and adsorbed by vegetation and the permeable surface, and a small part will be washed away with the stormwater runoff, which will contribute to pollution. The pollutant concentrations from greenbelt runoffs are presented in Table 5. Note, however, that the volume of stormwater runoff from a unit area of greenbelt may be orders of magnitude lower than those from the road and rooftop due to stormwater infiltration and absorption.

### 2.2. Methods

#### 2.2.1. Toughreact

During the process of infiltration in a managed aquifer recharge project, pollutants and porous media are used. The adsorption in the process of recharging can be simulated by TOUGHREACT, a numerical simulation program that determines chemically reactive non-isothermal flows of multiphase fluids in porous media. The program was written in Fortran77 and developed by introducing reactive chemistry into the framework of the existing multi-phase fluid and heat flow simulator TOUGH2 [77]. The program can be applied to one-, two-, or three-dimensional porous media with physical and chemical heterogeneity. The transportation of aqueous and gaseous species by advection and molecular diffusion is considered in both the liquid (aqueous) and gas phases [78]. In order to simulate the adsorption process, the adsorption kinetic reaction module was added to the program, and the modified one-dimensional convection–dispersion equation was as follows [79]:

(1) $$\frac{\partial C}{\partial t}=D\frac{\partial^{2}C}{\partial z}-v\frac{\partial C}{\partial z}-r,$$

where *C* is the initial concentration of a contaminant, mol/kg (mol of ion/kg of solution); *D* is the diffusion coefficient in ${\mathrm{cm}}^{2}/\min$; and v is the average pore flow rate in cm/min. z is the vertical distance in cm, t is the time in min, and r is the adsorption rate of the solution, which can be determined by the following equation:

(2) $$r=\frac{\rho_{b}}{n}\frac{\partial S}{\partial t}=k\psi C,$$

where $\rho_{b}$ is the density of the medium, g/cm^3^; n is the porosity (−); k is a first-order rate constant measured in min^−1^; and ψ is the function of ion deposition, which is determined by the following equation [80]:

(3) $$\psi =1-\frac{S}{S_{max}},$$

where S is the solid phase concentrations of attached ions in mol/kg (mol of ion/kg of sand), $S_{max}$ is the maximum solid phase concentrations of attached ions in mol/kg (mol of ion/kg of sand).

#### 2.2.2. Clogging Indicators

Since physical clogging mainly results from TSSs contained in source water, the concentration of TSSs is strictly controlled below 5 mg L^−1^. Additionally, for turbidity, it is suggested that its value should be controlled below 5 NTU to avoid clogging [81]. The membrane filtration index (MFI) is a common method used to assess the physical clogging potential that is caused by suspended particles in recharge water [82]—the greater value of the MFI, the higher the potential for physical clogging. A quantitative relationship between the clogging potential and the concentration of total suspended particles was set by Dillon (see Equation (4)) [83]:

(4) MFI=15.2×TSS,

where MFI represents the physical clogging potential index in $s/L^{2}$; TSS represents the concentration of suspended particles in mg/L; and 15.2 is the coefficient.

The chemical clogging indicator is the concentration of iron ions included, as iron ions can lead to precipitation with changes in redox conditions and pH and can occupy the pore volume to resist water flow, thereby causing clogging. 

Biological clogging indicators mainly depend on the concentration of nutrients, such as nitrogen and phosphorus, which can nourish the growth of bacteria.

## 3. Results and Discussion

### 3.1. Characteristics of the Urban Stormwater Quality

From the collected stormwater quality data, the pollutants in the stormwater were determined to be mainly nutrients, organic matter, total suspended solids or suspended solids (TSSs), and heavy metals. The nutrients were ammonia nitrogen (NH_3_-N), total nitrogen (TN), and total phosphorus (TP). The main indicator of organic matter was the chemical oxygen demand (COD). Heavy metal ions are the most common, especially zine, lead, copper, and cadmium. Iron and manganese were also detected in some areas. It should be noted that these conclusions are based on the data collected. Other possible contaminants, such as petroleum hydrocarbons (TPH), bacteria, viruses, and other trace elements, have not been consistently reported in the literature and are not discussed in this manuscript.

#### 3.1.1. Physicochemical Stormwater Properties

The physicochemical characteristics of stormwater from the road, roof, and green land were analyzed as follows.

##### Road Stormwater Quality

Table 6 shows a comparison with the surface water standard of Chinese Environment Quality Standards for Surface Water (CEQSSW, GB3838-2002, State Environment Protection Administration of China) [84], which was developed to strengthen management of the surface water environment and prevent water pollution. The basic surface water environment quality standard is applicable to surface water, with functions designated to uses in rivers, lakes, canals, and reservoirs. The water quality is distinguished into five grades (I, II, III, IV, V). Water quality below grade III is usually regarded as polluted. It is obvious that the concentration of collected pollutants exceeds the standard to varying degrees. The median concentration of COD is 152.91 mg L^−1^, almost seven times the standard value. Similarly, nutrients, including TN, NH_3_-N, and TP, all exceed the grade III water standard value by five, three, and two times, respectively. The major heavy metal pollutants from urban road runoff are zinc, lead, copper, and cadmium, with traces of iron and manganese. It is noted that Fe (1.85 mg L^−1^) has the highest mean value, around 10 times higher than the other metals. The median concentrations of Zn (0.34 mg L^−1^), Pb (0.04 mg L^−1^), Cu (0.04 mg L^−1^), and Cd (0.005 mg L^−1^) do not exceed the water grade III standard value of CEQSSW, whereas, in some areas, the maximum concentration of metals surpasses the standard value to a certain extent. In general, TN, Zn, Pb, and Cu always result from atmospheric and wet deposition and emissions from motor vehicle exhaust. The worn tire scrap and road material particles contribute to contamination with TP [23].

##### Roof Stormwater Quality

Table 7 clearly shows that the pollution degree of roof stormwater is less than that of road stormwater. The contamination resources of roof runoff include: (1) Atmospheric depositions, which includes dry depositions on sunny days and wet depositions caused by rain leaching during rainfall. This means that contaminants, such as N, P, and metals, in the atmosphere can accumulate on the roof surface by sedimentation. However, these contaminants are brought into the stormwater runoff by the leaching of rainfall. (2) The roof stormwater quality has a strong relationship with roof materials [62].

For different roof materials, the pollution degree varies. The median concentration of COD in asphalt roof stormwater is 85.14 mg L^−1^, which is nearly four times the standard value. TN and TP on asphalt roof are relatively higher than those of other nutrients, with median values of 9.51 and 0.17 mg L^−1^, respectively, whereas, the concentration of NH_3_-N in tile roof runoff is higher, nearly 3.5 times the standard value. The concentration of heavy metal ions in cement roof runoff is relatively higher, except for Zn. The mean concentrations of Pb, Cu, and Cd are 0.19, 0.044, and 0.025 mg L^−1^, respectively. However, the heavy metal concentration is below the standard value III, except in individual areas. Based on the analysis above, it is obvious that roofing materials have a very significant impact on the quality of stormwater runoff. For different roof materials, the pollutant concentration varies widely; for the same type of pollutant, the concentration changes depending on the roof material. 

##### Greenbelt Stormwater Quality 

TSSs, COD, TN, NH_3_-N, and TP are the major pollutants of stormwater runoff from the greenbelt. As shown in Table 8, the mean concentration of TSSs is 194.33 mg L^−1^, and the mean concentration of COD is 57.22 mg L^−1^, exceeding the standard value by almost two times. As for nutrients, such as TN, NH_3_-N, and TP, the pollution degree is serious, as the nutrient concentrations are worse than the standard value to a great extent.

### 3.2. Pollution Risk Analysis

Chemical pollutants, such as heavy metals and nutrients, can infiltrate into aquifers during MAR, which will potentially influence the groundwater quality. At the same time, physical adsorption between pollutants and porous media can occur to a certain degree. Moreover, the solute transport of chemical pollutants can be simulated by TOUGHREACT, which is an approach to simulate the adsorption of pollutants to porous media during the MAR process. 

**Figure 1:**
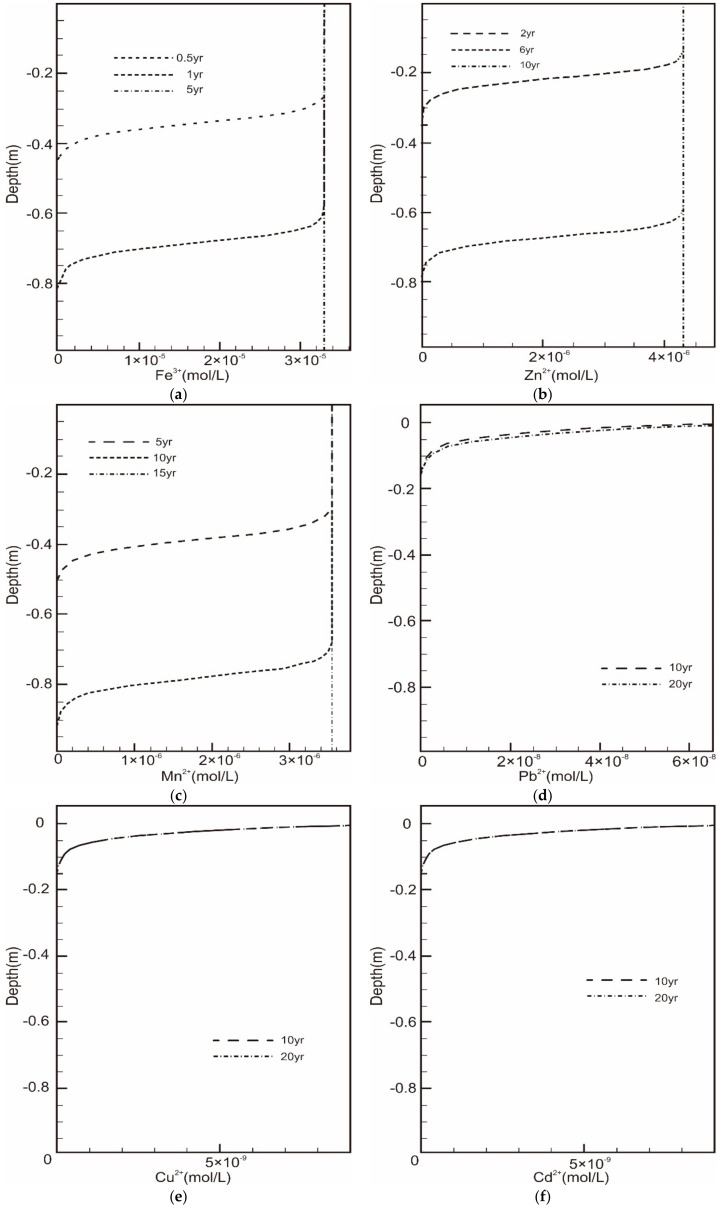

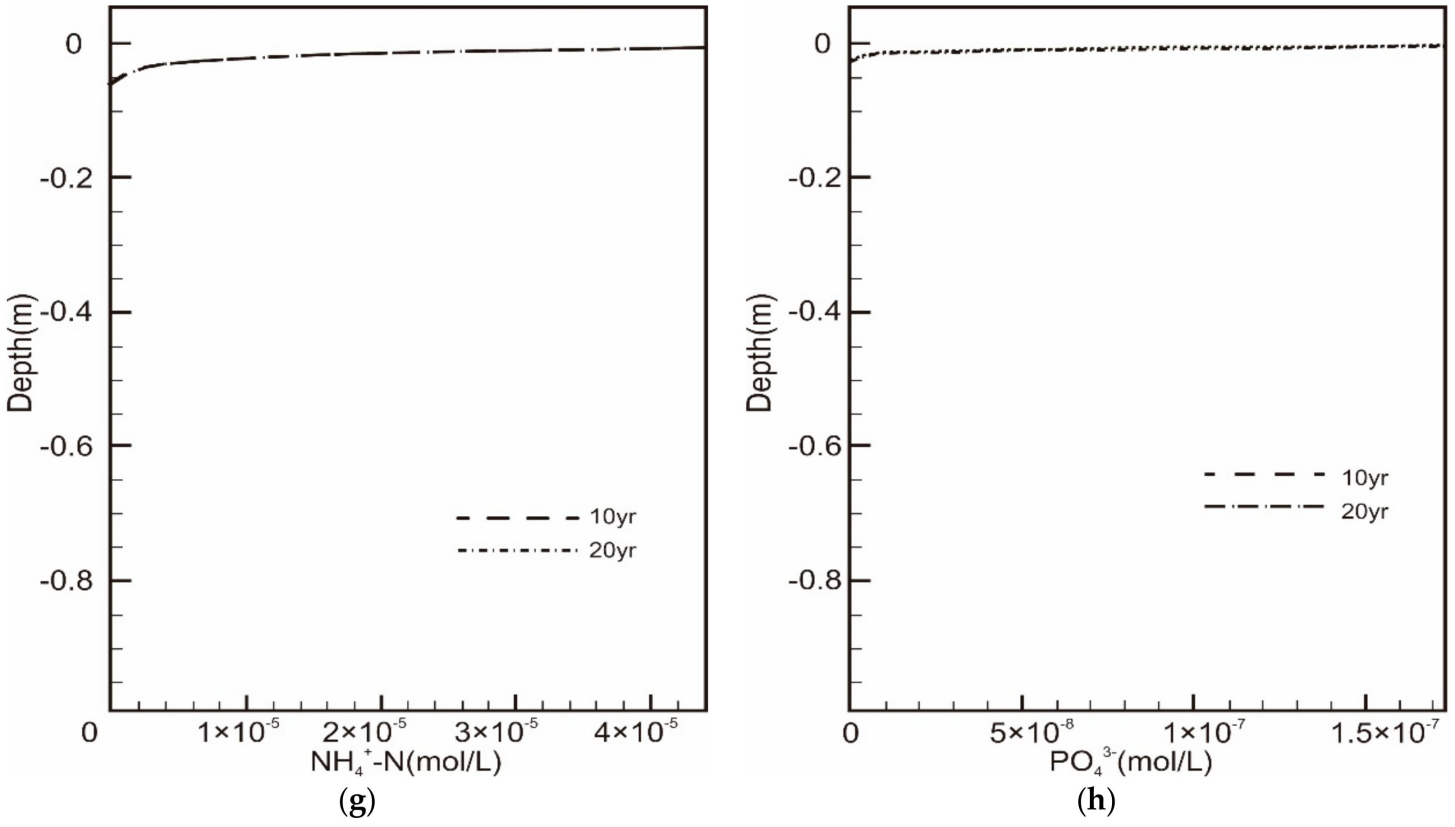
Results of chemical pollutant transport in the column. (**a**), (**b**), (**c**), (**d**), (**e**), (**f**), (**g**) and (**h**) were the pollutants’ infiltration curve for Fe^3+^, Zn^2+^, Mn^2+^, Pb^2+^, Cu^2+^, Cd^2+^, NH_4_^+^-N and PO_4_^3−^, respectively.

In order to quantitatively analyze the pollution risk of different substances, the 1D radial model was adopted. The amount of quartz sand per unit length of the infiltration medium was assumed to be homogenous initially (Figure 1). The -pollutant with the fastest migration rate had the higher risk of groundwater pollution. 

The inlet and outlet were conceptualized as the Neuman boundary for constant flow. The parameters for adsorption simulation are listed in Table 9. The initial concentrations of chemical pollutants were the median values of the collected data (Table 10).

The infiltration of various ions in the sand column is shown (in Figure 1).

The transport of chemical pollutants is influenced at different levels by adsorption between ions and the porous media, leading to migration rate diversity among chemical ions. Therefore, the time that chemical pollutants reach into the aquifer is delayed and the amount of pollutants is reduced due to adsorption in the vadose zone. It is obvious that the iron ion migrates rapidly, taking 5 years to saturate the whole sand column, followed by zinc and manganese, with migration times two-fold and four-fold as long as iron, respectively. The other ions, such as copper and lead, have lower rates of migration, leading to accumulation in the first 0 to 0.6 m. For nutrients, phosphorus is less mobile than nitrates in the vadose zine and is effectively removed through ion exchange. The results above coincide with previous studies [87]—the longer the migration time, the lower the contamination risk. Thus, using a five-year migration time through a 1 m sand column, the chemical ions can be divided into four according to the contamination risk (Table 11).

Based on the above analysis, it is suggested that before recharge with urban stormwater, the removal of chemical ions is of great importance. 

### 3.3. Clogging Risk

Clogging is recognized as perhaps the most significant challenge in MAR operations; it is attributed to physical, chemical, and biological processes.

#### 3.3.1. Physical Clogging

Physical clogging is the result of the accumulation or injection of organic and inorganic suspended solids blocking the pores of the infiltration. When the pore size of the infiltration media is larger than the diameter of the TSSs, some particles may penetrate to greater depths. As TSSs are deposited, however, they build up a restricting layer, which can limit flow. Besides, physical clogging caused by deposition of TSSs is the predominant cause of infiltration reduction [88]. The membrane filtration index (MFI) indicator can determine the potential for clogging. 

For the concentration of TSSs, its limitation is below 10 mg L^−1^ to avoid physical clogging [89]. However, regardless of the type of stormwater, the TSSs concentration exceeds the limit by a large margin. The median concentration of road stormwater is 439 mg L^−1^, followed by greenbelt stormwater (110.01 mg L^−1^) and roof stormwater (56 mg L^−1^).

Specifically, the clogging rate of the suspended matter permeating through the filter membrane with a diameter at 0.45 μm was determined. Based on the equation, the clogging risk of MAR was evaluated using urban stormwater. The value of MFI was calculated by the median concentration of TSSs (Table 6, Table 7 and Table 8) in different types of stormwater. The MFI values for road, roof, and green land are 8940.79, 1934.96, and 2953.82 $s/L^{-2}$, respectively. According to Dillon’s research, the MFI value at 110 $s/L^{-2}$ is regarded as having high clogging potential [83]. Considering both the MFI and TSSs concentrations, the physical clogging potential of different types of stormwater is very high, of which road stormwater has the highest clogging risk, followed by greenbelt and roof stormwater. 

#### 3.3.2. Biological Clogging

Biological clogging is affected by two factors: Biological growth and the accumulation of by-products resulting from the decomposition of biological products. The first factor, the biological growth, for example, algae and bacterial growth, can reduce the size of pores [88,90,91]. The second factor, by-products from biological degradation, such as cell bodies, produce bacterial extra-cellular polymers (e.g., polysaccharides), which can reduce the permeability of porous media, entrap gases (e.g., methane, nitrogen, and carbon dioxide) that increase resistance to liquid flow, and microbially catalyze the accumulation of insoluble precipitates [92,93,94]. Usually, biological clogging follows the physical changes but happens later. With the accumulation of suspended matter in the filtration medium, biological growth often takes place subsequently under favorable conditions and with adequate availability of nutrients. Researchers identified that the predominant bacterial causes of clogging include aerobic bacteria [95], denitrifying bacteria [96,97,98], acetogenic bacteria [95], sulfate-reducing bacteria [99,100], meth-anogenic bacteria [96,101], and photosynthetic sulfur bacteria [101]. Although the relevant microbiology data are not included in the collected data from the literature, some types of bacteria, such as *Pseudomonas aeruginosa*, have been proven to widely exist in urban stormwater and soil [102,103,104,105]. Once the nutrient is appropriate, the biological clogging risk will be induced. Carbon and nitrogen are two main nutrient resources for microbial growth. Carbon is represented by dissolved organic carbon (DOC) or total organic carbon (TOC) [106,107]. Nutrients in water, like nitrogen, support bacterial metabolism and the growth of algae, which can accelerate the clogging process. Therefore, the total nitrogen concentration can be used as a key index to evaluate biological clogging in aquifers. It is suggested that the concentration of total nitrogen (below 0.3 mg L^−1^) can decrease the biological clogging potential [108,109]. According to Table 6, Table 7 and Table 8, the median concentrations for the total nitrogen concentrations of road, roof, and green land stormwater are 5.87, 6.05, and 2.86 mg L^−1^. It is obvious that all types of stormwater have a risk of biological clogging, of which roof stormwater has the highest clogging risk. Before infiltration, there are several methods that can be used to avoid clogging. For example, biological clogging can be alleviated by removing organic carbons and other nutrients (e.g., N and P) from source water via bio-infiltration. This can be accomplished by infiltration with granular activated carbon (GAC), which has a great affinity for both organic carbons and inorganic nutrients. Besides, disinfection with chlorine or other disinfectants can help to retard biological clogging [110]. 

#### 3.3.3. Chemical Clogging

Chemical clogging usually occurs at the aquifer due to complex chemical reactions, such as precipitation and/or dissolution of minerals, which result from the mixing of different compositions from resource water and groundwater [91,111], but this type of clogging has not been widely recognized as a major clogging mechanism as it often coincides with other types of clogging and may take a long time to develop [112]. Both pH and redox conditions are two key factors that influence chemical clogging. Precipitation is often induced by an increase in pH as a result of algal activities, since algae remove dissolved carbon dioxide via photosynthesis. Among the various precipitates, Fe (III) (hydr)oxides are the most dominant [112,113], and they are susceptible to redox conditions. For example, during the MAR infiltration process, the increase in dissolved oxygen concentration can lead to oxidation of ferrous ions to ferric ions, followed by subsequent precipitation, which can occupy the pore volume and resist water flow [112,114]. Past research shows that even an iron concentration of 0.3 mg L^−1^ can lead to a severe reduction in permeability [32]. Based on the collected stormwater quality data, only iron ions exist in road stormwater, with a median concentration of 1.85 mg L^−1^, which leads to a high risk of chemical clogging. The risk of chemical clogging of roof and green land stormwater infiltration is low, at least for iron precipitation. 

The analysis above shows that clogging problems will inevitably exist when stormwater is employed as recharge water in MAR. To decrease the clogging risk, the recommended values for basic parameters are shown in Table 12.

## 4. Conclusions

The components of stormwater can contaminate groundwater and cause clogging of aquifer materials, thereby shortening the lifespan of MAR systems or requiring the MAR systems to be maintained frequently at high costs. In this study, the characteristics of stormwater data and the factors that influence stormwater quality were analyzed. The results show that the concentrations of TSSs, nutrients (TN, NH_3_-N, TP), and COD in all of stormwater indicate a water standard value of less than grade III according to CEQSSW to different extents. In terms of metals, only a few samples in some areas were shown to exceed the limit value. In addition, it is true that there are other contaminants, such as hydrocarbons, that exist in stormwater [116]. However, the quantity of detected contaminants is limited and is not applicable for data analysis. 

For the purpose of developing a sustainable recharge system, the pollution and clogging risks of MAR with stormwater were evaluated. The risk of contamination was divided into four types based on the different migration times of ions through the sand column, of which the iron ion has the highest risk of contamination, followed by Zn and Mn. The contamination risks of nutrients and other metals (Pb, Cu, and Cd) are relatively low.

The physical, biological, and chemical clogging risks were evaluated. The values of TSSs and MFI from different underlying surfaces were compared, indicating that the physical clogging potential of all of stormwater is very high. The biological clogging potential is relatively high because the concentration of TN is greater than the recommended value of 0.3 mg L^−1^. The risk order of biological clogging for stormwater is greenbelt stormwater < road stormwater < roof stormwater. Chemical clogging is mainly due to precipitation of iron in the process of MAR. In the collected data, iron was only found to exist in road stormwater, causing a higher chemical clogging risk. For roof and greenbelt stormwater, the chemical clogging risk was shown to be rather low. 

By the data retrieved from the literature, the study only analyzed the pollution and clogging risks of major pollutants in urban stormwater in the process of MAR, and the risk of contamination by other micro-polluting substances (such as bacteria, viruses, Pharmaceutical and Personal Care Products, PPCPs) and the risk of clogging by algae and other substances (e.g., Dissolved Organic Carbon, DOC) were not taken into consideration; the clogging risk in managed aquifer recharge is limited due to the practical difficulties and uncertainties based on various factors, which are not accounted for in a controlled study. In addition, for the prediction of pollution risk, only the assumed condition was used for analysis, and the influence of groundwater burial and hydrogeochemical conditions (such as pH, Eh, DO, etc.) were not considered. 

The above factors need to be further discussed in the context of particular sites. 

## Figures and Tables

**Table 1:** Stormwater quality of roads in various cities (mg L^−1^). COD: chemical oxygen demand; TN: total nitrogen; TP: total phosphorus; TSSs: total suspended solids.

City	TSS	COD	NH_3_-N	TN	TP	References
Beijing	53.45	67.52	2.9	5.88	0.2	[23]
Weifang	-	62.67	1.34	42.89	0.76	[24]
Xian	1020	-	7.9	45.4	0.4	[25]
Jinan	969.25	101.22	5.74	3.87	0.31	[26]
Chongqing	1730	76.3	3.7	-	0.24	[27]
Guangzhou	439	373	-	11.71	0.49	[28]
Hangzhou	152	-	0.68	5.58	0.109	[29]
Chengdu	801.17	484.48	-	3.92	0.12	[30]
Tianjin	524	132	7.65	12.37	0.89	[31]
Changchun	-	9.14	0.97	-	-	[32]
Chaoyang	-	30	1.21	1.71	-	[33]
Baoji	179.70	120.04	2.89	5.85	0.448	[34]
Handan	357.72	264.29	7.33	10.42	0.93	[35]
Haikou	244	114.2	-	0.95	0.13	[36]

**Table 2:** Urban pavement runoff heavy metal concentration (mg L^−1^).

City	Zn	Pb	Cu	Cd	Fe	Mn	References
Beijing	0.027	0.003	0.027	-	-	-	[23]
Guangzhou	2.06	0.115	0.16	0.0016	-	0.348	[28]
Nanjing	0.56	0.053	0.101	0.0014	0.463	-	[37]
Changchun	0.367	-	0.023	-	1.098	-	[32]
Weifang	0.02	-	0.01	-	-	-	[24]
Shanghai	0.06	0.00029	-	0.001	-	0.01	[38]
Tianjin	-	-	0.011	0.010	-	-	[39]
Urumchi	1.390	1.954	0.322	0.0058	-	-	[40]
Lulea	0.15	0.0166	-	0.0383	-	-	[41]
Paris	0.55	0.133	0.061	0.0006	-	-	[42]
Texas	0.1528	0.0112	-	0.0239	1.491	-	[43,44]
California	-	0.017	-	0.0094	-	-	[45]
Ohio	0.459	0.037	0.043	0.005	4.136	0.324	[46]
Maryland	1.18	0.22	0.11	0.035	-	-	[47]
Los Angeles	0.506	0.033	0.931	0.0025	-	-	[48]
Otsu	0.268	0.014	0.036	0.0002	-	0.066	[49]
Nantes, France	0.32	0.057	0.036	0.0013	-	-	[50]
Weins	0.441	0.137	0.049	0.0009	2.2	-	[51]
Christchurch	0.102	0.007	0.026	-	-	-	[52]
Genoa	0.081	0.013	0.019	-	-	-	[53]
Ghaha	0.678	0.409	0.343	0.035	2.31	-	[54]

**Table 3:** The concentration of roof runoff pollutants (mg L ^−1^).

Cities	Roof Type	COD	TSS	TN	TP	NH_3_-N	References
Jiangbei, District, Chongqing	tile	39	26	3.70	0.09	1.00	[58]
Yubei District, Chongqing	tile	48.10	37.00	4.03	0.12	1.19	[59]
Yubei District, Chongqing	tile	147.00	61.70	4.23	0.35	1.46	[60]
Chongqing University	tile	64.30	700.00	-	0.07	5.60	[27]
Wuhan Wulidun	tile	46.00	16.30	1.98	0.08	-	[61]
Nanjing	tile	39.80	77.40	5.80	0.27	-	[62]
Beijing	tile	123	136.00	-	-	-	[42]
Tianjin	tile	99	249	11.92	0.12	6.37	[31]
Weifang	tile	35.97	-	1.79	0.71	29.39	[24]
Harbin	asphalt	111.8	110.3	-	0.21	1.37	[63]
Yubei District, Chongqing	asphalt	44.70	8.30	3.63	0.06	1.36	[60]
Chongqing University	asphalt	74.30	200.00	-	0.16	2.70	[27]
China Academy of Science, Bejing	asphalt	68.91	37.70	11.75	0.08	5.67	[23]
Middle layer, Tsinghua University	asphalt	95.97	34.36	11.81	0.09	7.56	[64]
High layer, Tsinghua University	asphalt	341.27	39.32	25.25	0.11	14.73	[64]
Nanjing	asphalt	51.30	50.20	7.30	0.24	-	[62]
Wuhan, Wulidun	asphalt	61.50	46.70	4.18	0.34	-	[61]
Beijing	asphalt	328.00	136.0	9.80	0.94	-	[42]
Tianjin	asphalt	126	452	10.75	0.18	7.69	[31]
Development, Hangzhou	asphalt	3.62	33.6	3.69	0.077	0.18	[29]
Handan	asphalt	277.50	329.10	6.97	9.90	0.85	[35]
Yubei District, Chongqing	cement	77.50	61.70	6.20	0.12	1.03	[59]
Jiangbei, District, Chongqing	cement	68	56	5.9	0.15	1.85	[58]
Haikou	cement	44.80	63.0	1.00	0.04	-	[36]
Wuhan, Wilimiao	cement	78.90	49.70	2.43	0.09	-	[61]
Nanjing	cement	49.00	47.20	7.00	0.19	-	[62]
Beijing	cement	115.99	27.00	8.26	0.71	-	[65]
Shandong	cement	85.05	351.5	6.27	0.16	6.82	[26]

**Table 4:** The concentration of metals in roof runoff.

Cities	Roof Type	Zn	Pb	Cu	Cd	References
Beijing	tile	0.408	0.026	-	0.0029	[66]
Weifang	tile	35.97	-	0.02	-	[24]
Nanjing	tile	0.022	0.01	-	0.00019	[67]
Yubei District, Chongqing	tile	0.0175	0.0048	0.006	0.0006	[59]
Beijing	asphalt	0.28	0.023	-	0.0015	[66]
China Academy of science, Bejing	asphalt	0.063	0.017	0.012	0.00007	[68]
Nanjing	asphalt	0.16	0.001	0.0075	0.00022	[69]
Yubei District, Chongqing district	cement	0.0328	0.0035	0.0089	0.0006	[59]
Macao	cement	0.029	0.0047	-	-	[70]
Jiangbei District, Chongqing	cement	0.32	0.564	0.08	0.05	[58]

**Table 5:** The concentration of greenbelt stormwater runoff (mg L^−1^).

Cities	TSS	COD	TN	TP	NH_3_-N	References
Water research institute, Beijing	-	-	2.56	0.15	1.05	[71]
Water research institute, Beijing	95	8.7	-	-	3.95	[72]
Chongqing	650	23	-	0.21	1.6	[27]
Haikou	127	38.2	0.68	0.2	-	[36]
Dongguan	87.42 (TSS)	67.94	2.42	0.89	0.85	[73]
China Academy of Science, Beijing	-	120.37	6.80	0.74	-	[74]
Xian	76.23	53.27	5.3	0.57	3.7	[25]
Ningbo	321.2	56.05	0.856	0.894	0.336	[75]
Kunming	72.8	55	2.80	0.38	2.73	[76]
Handan	125.02	92.50	4.69	0.51	3.30	[35]

**Table 6:** Summary of the physicochemical characteristic of road stormwater quality (mg L^−1^).

Variables	Road Stormwater Quality			
	Median	Minimum	Maximum	Standard *
TSS	439.00	53.45	1730.00	-
COD	107.71	9.14	484.48	20
TN	5.87	0.95	45.40	1.0
NH_3_-N	2.90	0.68	7.90	1
TP	0.36	0.11	0.93	0.2
Zn	0.34	0.02	2.06	1
Pb	0.04	0.0003	1.95	1
Cu	0.04	0.01	0.93	1
Cd	0.005	0.0002	0.0383	0.005
Fe	1.85	0.463	4.14	-
Mn	0.20	0.01	0.35	-

* The water grade III standard value of CEQSSW.

**Table 7:** Summary of physicochemical characteristic of roof stormwater quality.

Variables	Units	Tile Roof Runoff Quality			Asphalt Roof Runoff Quality			Cement Roof Runoff Quality			Standard *
		Med	Min	Max	Med	Min	Max	Med	Min	Max	
TSS/SS	mg L^−1^	69.55	16.3	700	48.45	8.30	452.0	56.00	27.00	351.5	-
COD	mg L^−1^	48.10	35.97	147.00	85.14	3.62	314.27	77.50	44.80	115.99	20
TN	mg L^−1^	4.03	1.79	11.92	8.55	3.63	25.25	6.20	1.00	8.26	1.0
NH_3_-N	mg L^−1^	3.53	1.00	29.39	2.70	0.18	14.73	1.85	1.03	6.82	1
TP	mg L^−1^	0.12	0.07	0.71	0.17	0.06	9.9	0.15	0.04	0.71	0.2
Zn	mg L^−1^	0.215	0.018	35.97	0.16	0.028	0.063	0.032	0.029	0.32	1
Pb	mg L^−1^	0.01	0.005	0.026	0.017	0.001	0.023	0.005	0.004	0.564	1
Cu	mg L^−1^	0.013	0.006	0.02	0.010	0.00752	0.012	0.044	0.009	0.08	1
Cd	mg L^−1^	0.0006	0.0002	0.0029	0.0002	0.0001	0.0015	0.025	0.0006	0.05	0.005

- not given. * The water grade III standard value of the CEQSSW.

**Table 8:** Summary of the physicochemical characteristic of green land stormwater quality.

Variables	Units	Greenbelt Runoff Quality			Standard *
		Med	Min	Max	
TSS	mg L^−1^	110.01	72.80	650.00	-
COD	mg L^−1^	55.00	8.70	120.37	20
TN	mg L^−1^	2.68	0.68	6.80	1.0
NH_3_-N	mg L^−1^	2.17	0.336	3.95	1
TP	mg L^−1^	0.51	0.15	0.89	0.2

- not given. * The water grade III standard value of CEQSSW.

**Table 9:** Parameters of chemical pollutants for adsorption.

Ions	$k\;(\min^{-1})$	$S_{max}\;(\mathrm{mol}/\mathrm{kg})$	References
Pb^2+^	4.00 × 10^−4^	3.48 × 10^−4^	[85,86]
Zn^2+^	1.16 × 10^−3^	2.96 × 10^−4^	
Cu^2+^	9.84 × 10^−4^	3.98 × 10^−4^	
Cd^2+^	8.64 × 10^−3^	5.75 × 10^−4^	
Fe^3+^	9.60 × 10^−4^	3.60 × 10^−4^	
Mn^2+^	9.00 × 10^−4^	3.50 × 10^−4^	
NH_4_^+^-N	3.14 × 10^−2^	1.624	
PO_4_^2−^	4.08 × 10^−2^	0.055	

k is the adsorption rate constant, $S_{max}$ is the maximum adsorption concentration.

**Table 10:** Initial concentration of chemical ions.

Ions	Zn^2+^	Pb^3+^	Cu^2+^	Cd^2+^	Fe^3+^	Mn^2+^	NH_4_^+^-N	PO_4_^3−^
Concentration(mol/kg)	4.3 × 10^−6^	8.2 × 10^−8^	5.6 × 10^−7^	1.4 × 10^−8^	3.3 × 10^−5^	3.5 × 10^−6^	9.2 × 10^−5^	2.2 × 10^−6^

**Table 11:** Summary of the migration time for each chemical ion.

	Ions	Fe^3+^	Zn^2+^	Mn^2+^	Cd^2+^, Pb^2+^, Cu^2+^, NH_4_^+^-N, PO_4_^3−^
Items					
Migration time (year)		5	10	15	>20
Risk rating		IV	III	II	I

**Table 12:** Recommended values for basic parameters. MFI: membrane filtration index.

Types	Pollutants	Limitations	References
Physical clogging	TSS	TSS < 10 mg L^−1^	[79]
		MFI < 110 s L^−2^	[89]
Biological clogging	Bacteria	TN < 0.3 mg L^−1^	[109]
	Algae		
Chemical clogging	Iron	Fe < 0.3 mg L^−1^	[32,115]
